# The Impact of Utilizing Different Optical Coherence Tomography Devices for Clinical Purposes and in Multiple Sclerosis Trials

**DOI:** 10.1371/journal.pone.0022947

**Published:** 2011-08-11

**Authors:** Christina V. Warner, Stephanie B. Syc, Aleksandra M. Stankiewicz, Girish Hiremath, Sheena K. Farrell, Ciprian M. Crainiceanu, Amy Conger, Teresa C. Frohman, Esther R. Bisker, Laura J. Balcer, Elliot M. Frohman, Peter A. Calabresi, Shiv Saidha

**Affiliations:** 1 Department of Neurology, Johns Hopkins University School of Medicine, Baltimore, Maryland, United States of America; 2 Department of Biostatistics, Johns Hopkins University Bloomberg School of Public Health, Baltimore, Maryland, United States of America; 3 Department of Neurology, University of Texas Southwestern Medical Center, Dallas, Texas, United States of America; 4 Department of Neurology, University of Pennsylvania School of Medicine, Philadelphia, Pennsylvania, United States of America; National Institutes of Health, United States of America

## Abstract

Optical coherence tomography (OCT) derived retinal measures, particularly peri-papillary retinal nerve fiber layer (RNFL) thickness, have been proposed as outcome measures in remyelinating and neuroprotective trials in multiple sclerosis (MS). With increasing utilization of multiple centers to improve power, elucidation of the impact of different OCT technologies is crucial to the design and interpretation of such studies. In this study, we assessed relation and agreement between RNFL thickness and total macular volume (in MS and healthy controls) derived from three commonly used OCT devices: Stratus time-domain OCT, and Cirrus HD-OCT and Spectralis, two spectral-domain (SD) OCT devices. OCT was performed on both Cirrus HD-OCT and Stratus in 229 participants and on both Cirrus HD-OCT and Spectralis in a separate cohort of 102 participants. Pearson correlation and Bland-Altman analyses were used to assess correlation and agreement between devices. All OCT retinal measures correlated highly between devices. The mean RNFL thickness was 7.4 µm lower on Cirrus HD-OCT than Stratus, indicating overall poor agreement for this measurement between these machines. Further, the limits of agreement (LOA) between Cirrus HD-OCT and Stratus were wide (−4.1 to 18.9 µm), indicating poor agreement at an individual subject level. The mean RNFL thickness was 1.94 µm (LOA: −5.74 to 9.62 µm) higher on Spectralis compared to Cirrus HD-OCT, indicating excellent agreement for this measurement across this cohort. Although these data indicate that these three devices agree poorly at an individual subject level (evidenced by wide LOA in both study cohorts) precluding their co-utilization in everyday practice, the small difference for mean measurements between Cirrus HD-OCT and Spectralis indicate pooled results from these two SD-devices could be used as outcome measures in clinical trials, provided patients are scanned on the same machine throughout the trial, similar to the utilization of multiple different MRI platforms in MS clinical trials.

## Introduction

Although multiple sclerosis (MS) is conventionally regarded as an immune-mediated demyelinating disorder of the central nervous system (CNS), neuroaxonal degeneration represents a significant proportion of MS pathobiology, and has been shown to be the major correlate of MS related disability [1]–[5]. While the etiology of axonal degeneration in MS remains incompletely elucidated, it is primarily thought to occur either as a result of acute, immune-mediated axonal transection or as a result of chronic demyelination with associated loss of trophic support [6]–[10]. Neuronal atrophy or loss in MS is in turn thought to be the derivative of retrograde axonal degeneration or anterograde transynaptic degeneration [11]–[13]. Since a major pathologic substrate of neuroaxonopathy in MS may be chronic demyelination, and neuroaxonopathy is regarded as the major determinant of disability in MS, remyelinating and neuroprotective strategies have become important therapeutic goals in MS. With increasing research of potential remyelinating and neuroprotective drugs, and the imminent transition of such agents from laboratory to clinical trial testing, tools allowing objective quantification of the effects of such strategies on myelin, neurons and axons are required. One such potential tool proposed for this purpose is optical coherence tomography (OCT) [14], [15].

OCT is a rapid, non-invasive, office-based imaging technique allowing objective quantification of retinal structures with high resolution, including determination of peri-papillary retinal nerve fiber layer (RNFL) thickness and total macular volume (TMV) [16]–[19]. The anterior visual pathway is a frequent target of the MS disease process. Acute optic neuritis (AON) occurs in approximately 30–70% of MS patients during the course of their illness and 94–99% of MS patients have plaques in their optic nerves at post-mortem analysis, irrespective of AON history [20]–[23]. Optic nerve demyelination (due to clinical or subclinical optic neuropathy) results in retrograde degeneration of constituent optic nerve axons. Since these axons originate from the retinal nerve fibers, this process is reflected by RNFL thinning (which may be objectively quantified by OCT) [24]. Retinal nerve fiber degeneration may in turn lead to death of ganglion cells (from which they originate) and contribute to reductions in TMV as measured by OCT [12], [25]. RNFL thickness has been shown to correlate with disability, brain atrophy, visual function and visual quality of life in MS [26]–[32].

Given the predilection of MS to afflict the optic nerves and the lack of confounding related to myelin (the axonal projections of retinal ganglion cells to the optic nerves are uniquely unmyelinated) [33], [34], the eye has been proposed as a model within which to study the neurodegenerative processes associated with MS [14], [15]. Demyelinated yet intact axons, could potentially be remyelinated or protected, resulting in RNFL and ganglion cell preservation. Several characteristics of OCT, including pathological specificity, good structure-function correlation, good reproducibility and reliability and its potential to identify change over time, enable its use to detect and monitor the course of disease-related neurodegeneration in MS and to document the neuroprotective and potentially neurorestorative effects of novel therapeutic agents [25]–[28], [35]–[38].

Within the past decade, the greatest advancement in OCT technology has been the development of fourth-generation spectral domain (SD) OCT. SD-OCT has faster axial scan velocities, higher axial resolution and better reproducibility than older third-generation time domain (TD) OCT technology [37], [39]–[42]. In addition to different generations of OCT technologies (with differing measurement algorithms), there are multiple OCT machines of the same generation produced by different manufacturers, with known differences in the measurement algorithms between these machines [43], [44]. While comparisons have been previously performed between various OCT machines [43]–[45], such comparisons have not specifically assessed agreement or compatibility of conventional OCT measures derived from differing OCT analysis platforms. If multi-center trials utilizing OCT outcome measures are to be performed, this information is crucial to the design and interpretation of these studies. We examined the agreement between measures of RNFL thickness and TMV obtained with Stratus TD-OCT (Carl Zeiss Meditec, Dublin, California), Cirrus High Definition (HD) SD-OCT(Carl Zeiss Meditec, Dublin, California), and Spectralis SD-OCT (Heidelberg Engineering, Heidelberg, Germany) in MS and healthy controls.

## Methods

The study protocol for this specific study was approved by the Institutional Review Boards of Johns Hopkins University and the University of Texas Southwestern, and the study was performed in accordance with the ethical standards laid down in the 1964 Declaration of Helsinki. Written informed consent was obtained from all study participants, who represented an unselected convenience sample of subjects willing to undergo evaluation for research purposes. MS participants had their diagnosis confirmed by the treating neurologist (P.A.C, E.A.F), based on McDonald criteria [46], and MS classification was recorded based on recognized MS subtypes [47] – relapsing remitting MS (RRMS), secondary progressive MS, primary progressive MS and relapsing progressive MS. The latter three progressive MS variants were combined as one group (progressive MS) in analyses due to the small sample sizes of these individual groups. Healthy controls were recruited from among Johns Hopkins University and the University of Texas Southwestern staff and unaffected family members of MS patients. Exclusion criteria for study participation included a history of ocular surgery, retinal disease, glaucoma, hypertension, diabetes, and spherical refractive error of more than 6.0 diopters. All OCT scans were performed without pupillary dilatation by experienced technicians.

In the Cirrus-Stratus cohort, scans were performed in random order on both Stratus OCT model 3000, software version 4.0.2 and Cirrus HD-OCT model 4000, software version 3.0 during the same clinical visit. Using the Stratus OCT device, RNFL thickness was acquired using the fast retinal thickness protocol consisting of three consecutive 3.4 mm-diameter circular scans (256 A-scans/B-scan) centered on the optic disc. The fast macular thickness protocol, which uses six 6-mm long intersecting radial scan lines centered on the fovea (128 A-scans/B scan), was used to calculate TMV. Cirrus HD-OCT RNFL thickness measurements were acquired using the optic disk cube 200×200 protocol. This protocol consists of 200 horizontal scan lines (each composed of 200 A-scans) that form a 6×6×2 mm volume cube from which a circle of 1.73 mm radius is automatically centered on the middle of the optic disc. Cirrus HD-OCT macular data were obtained using the macular cube 512×128 protocol (512 A-scans/ B-scan; the central vertical and horizontal scans are composed of 1024 A-scans), which forms a 6×6×2 mm volume cube. This scan protocol provides a profile of TMV, average macular thickness (AMT) and average thickness for the 9 macular subfields as defined by the Early Treatment Diabetic Retinopathy Study (ETDRS) areas [48]. Scans with signal strengths less than 7 were not included in our analyses.

In the Cirrus-Spectralis cohort, scans were performed in random order on both Cirrus HD-OCT and Spectralis OCT software version 5.2.4 on the same day. The Cirrus HD-OCT model and scan protocol are described above. Spectralis OCT RNFL thickness measurements were acquired using the RNFL-N protocol with an Automatic Real Time (ART) of 16 and a signal quality of at least 20 dB. Spectralis macular data were obtained using 20×20 degree (or greater) raster scans consisting of at least 25 lines each. The macular scans had an ART of at least 11 and a signal quality of at least 20 db. The TMV was calculated within the ETDRS macular grid.

Calculations and statistical analyses were performed using Stata 11.0 (StataCorp, College Station, TX). Only the right eyes of participants were used for analyses to avoid bias due to inter-eye correlation. T-test was used to compare OCT measurements between MS and healthy controls, as the examined variables followed a normal distribution. Pearson correlation coefficients were used to assess the linear relation between OCT-derived retinal measures from different OCT machines. The agreement between retinal measures acquired from different machines was evaluated with the Bland-Altman method [49], [50]. The interscanner agreement index was calculated for both RNFL thickness and TMV measurements for each subject. The interscanner agreement index, as defined by Bland and Altman has previously been used in the quantification of interscanner variation between MRI machines [51], [52], as it is common practice for different MRI analysis platforms to be co-utilized in MS trials [53]–[55].

If x_a_ is the measurement on machine a, and x_b_ is the measurement on machine b, then the interscanner agreement is defined as follows:
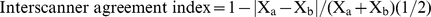
Stratus and Spectralis OCT calculate TMV from the sum of the 9 macular ETDRS subfields. Alternatively, Cirrus HD-OCT calculates TMV from a much larger area of the retina, the 6×6×2 mm volume cube. To more accurately compare TMV measures between the 3 OCT machines, we used the manufacturer's (Carl Zeiss Meditec) formula to calculate TMV from Cirrus HD-OCT over the 9 macular ETDRS subfields:
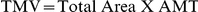


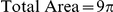


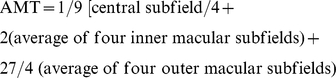



## Results

A total of 229 participants (RRMS: n = 138, mean age = 40.8±10.7; Progressive MS: n = 22, mean age = 52.3±10.0; Healthy controls: n = 69, mean age = 35.2±9.0) were included in the Cirrus HD-OCT vs. Stratus OCT part of this study. Of these, 174 were women and 55 were men, and there were no significant differences in sex ratios between the groups. Summary of statistical analyses from this cohort are illustrated in Table 1.

**Table 1:** Retinal Nerve Fiber Layer Thicknesses and Total Macular Volume Among Cirrus HD-OCT vs. Stratus Study Participants.

	Stratus OCT	Cirrus-HD OCT	r	*P value*	Mean Difference (95% CI)	Lower LOA (95% CI)	Upper LOA (95% CI)	Range
RNFL Thickness*								
All Participants (n = 229)	95.7±15.9	88.3±13.8	0.93	<0.001	7.4 (6.7 to 8.2)	−4.1 (−5.4 to −2.8)	18.9 (17.6 to 20.2)	23.00
RRMS (n = 138)	92.8±16.0	85.7±14.3	0.93	<0.001	7.1 (6.1 to 8.1)	−4.4 (−6.1 to −2.7)	18.6 (16.9 to 20.3)	23.04
Progressive MS (n = 22)	88.5±15.9	82.8±12.0	0.93	<0.001	5.7 (2.9 to 8.5)	−7.0 (−11.6 to −2.3)	18.3 (13.7 to 23.0)	25.30
Healthy Controls (n = 69)	103.9±12.1	95.3±10.3	0.90	<0.001	8.7 (7.4 to 9.9)	−2.1 (−4.4 to 0.1)	19.4 (17.2 to 21.8)	21.52
TMV**								
All Participants (n = 229)	6.63±0.45	7.93±0.47	0.95	<0.001	−1.30 (−1.31 to −1.28)	−1.59 (−1.62 to −1.59)	−1.01 (−1.04 to −0.98)	0.58
RRMS (n = 138)	6.53±0.5	7.82±0.47	0.95	<0.001	−1.29 (−1.32 to −1.27)	−1.59 (−1.63 to −1.55)	−0.99 (−1.03 to −0.95)	0.60
Progressive MS (n = 22)	6.41±0.36	7.75±0.38	0.95	<0.001	−1.34 (−1.39 to −1.29)	−1.58 (−1.66 to −1.50)	−1.10 (−1.18 to −1.02)	0.48
Healthy Controls (n = 69)	6.89±0.35	8.20±0.37	0.93	<0.001	−1.31 (−1.34 to −1.27)	−1.59 (−1.65 to −1.53)	−1.03 (−1.09 to −0.97)	0.56

Abbreviations: OCT: Optical coherence tomography; RNFL: Retinal nerve fiber layer; RRMS: Relapsing remitting multiple sclerosis; MS: Multiple sclerosis; TMV: Total macular volume; CI: confidence interval; LOA: limit of agreement.

*All measurements are in µm.

**All measurements are in mm^3^.

**Figure 1:**
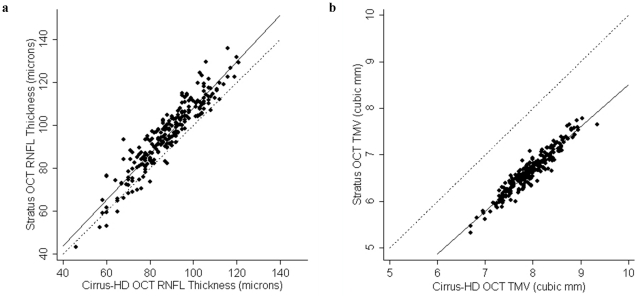
Correlation between RNFL thickness and TMV measures between Stratus OCT and Cirrus HD-OCT. Scatter plot of average RNFL thickness (1a) and TMV (1b) measured by Stratus OCT plotted against average RNFL thickness and TMV measured by Cirrus HD-OCT (n = 229). There was a strong linear agreement between Cirrus HD-OCT and Stratus OCT measurements of RNFL thickness (solid line) (r = 0.93; p<0.001) and TMV (solid line) (r = 0.95; p<0.001). The line of equality (dotted line) demonstrates Cirrus HD-OCT measurements of RNFL thickness are generally lower than with Stratus OCT, and Cirrus HD-OCT measurements of TMV are consistently greater than with Stratus OCT.

**Figure 2:**
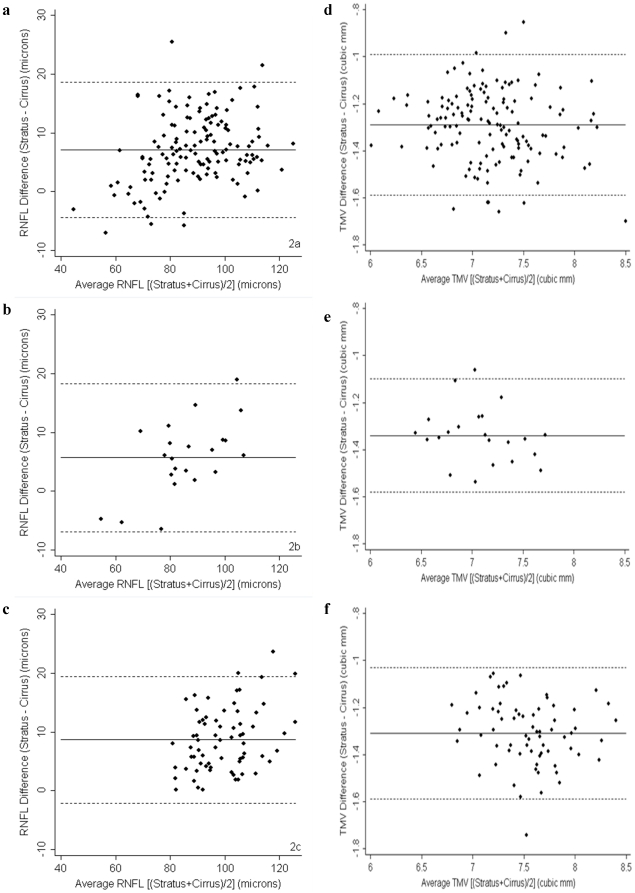
Agreement between Cirrus HD-OCT and Stratus OCT measures of RNFL thickness and TMV. Bland-Altman plots show poor agreement between Cirrus HD-OCT and Stratus OCT measures of RNFL thickness for RRMS (2a), progressive MS (2b) and healthy control (2c) eyes and suboptimal agreement between measures of TMV for RRMS (2d), progressive MS (2e) and healthy control (2f) eyes. Within each plot, the solid line indicates the mean RNFL thickness difference, while the dotted line denotes the 95% LOA. On average, Cirrus HD-OCT measured a lower RNFL thickness than Stratus OCT and the LOA were wide. On average, Cirrus HD-OCT measured a higher TMV than Stratus OCT, but with narrow LOA indicating the potential for agreement for this measurement at an individual subject level by extrapolation.

Cirrus HD-OCT RNFL thickness and TMV were significantly lower in RRMS and progressive MS than healthy controls (p<0.001 for both measures in both comparisons), in keeping with prior studies [25], [26], [28]. While Cirrus HD-OCT and Stratus OCT measures of RNFL thickness correlated strongly (r = 0.93; P<0.001) (Figure 1) with one another, Cirrus HD-OCT consistently measured lower average RNFL thickness than Stratus, where the mean difference was 7.4 µm (95% CI: 6.7–8.2 µm), indicating poor agreement on average for this measurement between the two machines across the cohort. The mean difference was determined using the Bland-Altman method [49], [50], and the difference in RNFL thickness measurements (Stratus RNFL−Cirrus RNFL) was plotted over mean RNFL thickness (Cirrus RNFL+Stratus RNFL/2) (Figure 2). Moreover, as the mean RNFL thickness increased, the calculated difference between the two devices also increased, resulting in wide 95% limits of agreement (LOA: −4.1 to 18.9 µm) spanning 23 µm, indicating poor agreement at an individual subject level (subgroup analyses presented in Table 1 and Figure 2).

Across the entire cohort, there was a strong correlation between the two machines for measures of TMV (r = 0.95; P<0.001) (Figure 1). Cirrus HD-OCT consistently measured a greater TMV than Stratus OCT (Figure 1), where the mean difference was −1.30 mm^3^ indicating less than optimal agreement for this measurement between the two devices across the cohort. The 95% LOA ranged from −1.59 mm^3^ to −1.01 mm^3^, which spanned 0.58 mm^3^ (Figure 2), indicating that one can achieve good agreement at an individual subject level by extrapolation (by adding a constant of 1.33 mm^3^ to Stratus measurements) (subgroup analyses presented in Table 1 and Figure 2).

A separate cohort of 102 participants (RRMS: n = 66; healthy controls: n = 36) were included in the Spectralis vs. Cirrus HD-OCT part of the study, in order to limit strain on our study population. Of these, 84 were women and 18 were men, and there were no significant differences in sex ratios between the groups. The mean age was 41.5±9.7 years in the RRMS group and 34.6±9.3 years in the healthy control group.

**Figure 3:**
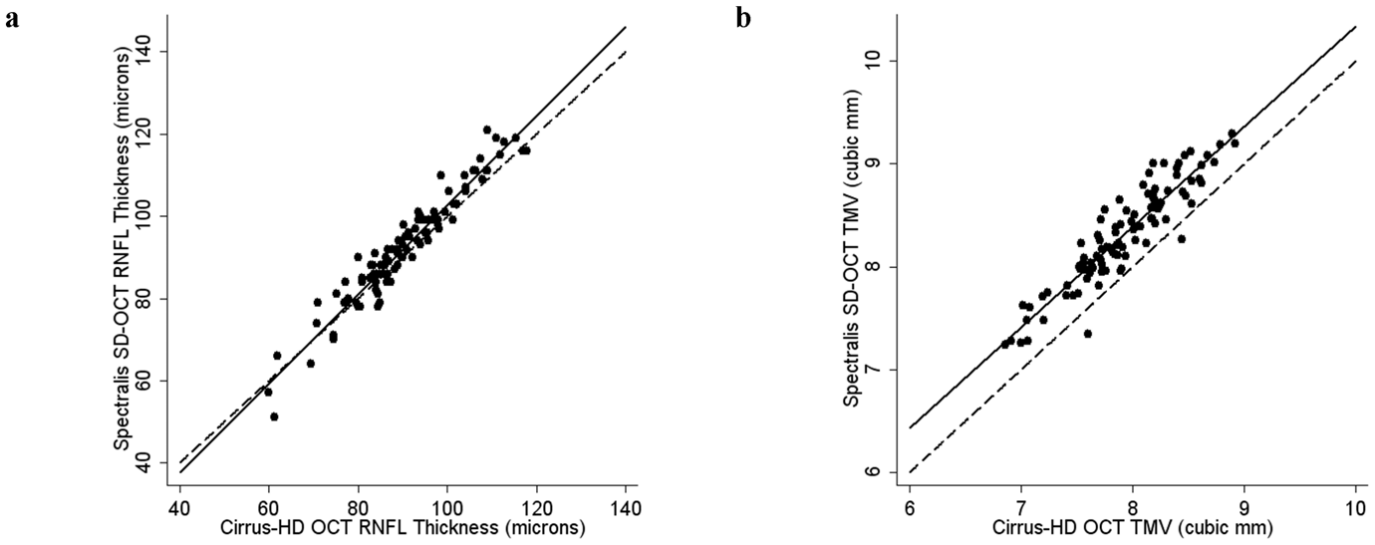
Correlation between RNFL thickness and TMV measures between Spectralis OCT and Cirrus HD-OCT. Scatter plot of average RNFL thickness (3a) and TMV (3b) measured by Spectralis OCT plotted against average RNFL thickness and TMV measured by Cirrus HD-OCT (n = 95). There was a strong linear agreement between Spectralis and Cirrus HD-OCT measurements of RNFL thickness (solid line) (r = 0.9622; P<0.001) and TMV (r = 0.9255; P<0.001). The line of equality (dotted line) demonstrates Spectralis measurements of RNFL thickness are slightly higher than with Cirrus HD-OCT, and that Spectralis measurements of TMV are also generally higher than with Cirrus HD-OCT.

**Figure 4:**
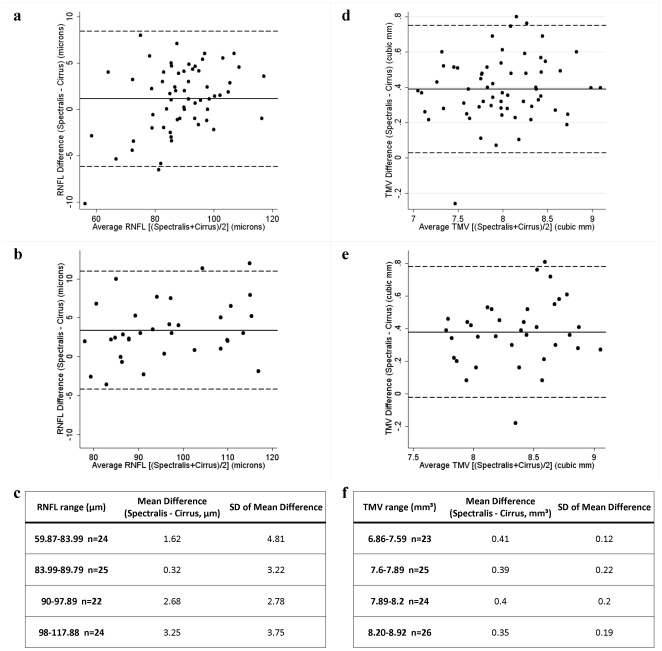
Agreement between Spectralis OCT and Cirrus HD-OCT measures of RNFL thickness and TMV. Bland-Altman plots show low mean differences (solid lines) indicating good agreement on average across studied cohorts, but with wide LOA (dotted lines) indicating poor agreement at an individual patient/subject level between Spectralis and Cirrus HD-OCT derived measures of RNFL thickness in RRMS (4a) and healthy control (4b) eyes and measures of TMV in RRMS (4d) and healthy control (4e) eyes. On average, Spectralis measured higher values of RNFL thickness and TMV than Cirrus HD-OCT. Analysis of the mean differences between Spectralis and Cirrus HD-OCT by quartile revealed consistent variability between machines with respect to RNFL thickness (4c) and TMV (4f) measures.

RNFL thickness was measured on both Spectralis and Cirrus HD-OCT machines in 95 participants (61 RRMS and 34 healthy controls), while TMV was measured on both devices in 98 participants (63 RRMS and 35 healthy controls). Summary of statistical analyses from this cohort are illustrated in Table 2. Spectralis RNFL thickness and TMV were significantly lower in RRMS than healthy controls (p<0.001 for both), in keeping with prior studies [25], [26], [28]. Overall, there was a strong correlation between Spectralis and Cirrus HD-OCT measures of RNFL thickness (r = 0.96; P<0.001), however the correlation line slightly overlapped the line of equality (Figure 3). Bland-Altman analyses revealed that Spectralis consistently measured higher average RNFL thickness than Cirrus HD-OCT, where the mean difference was 1.94 µm (95% CI: 1.15–2.72 µm), indicating excellent agreement on average for this measurement between the two devices across the cohort (Figure 4). However, as the mean RNFL thickness increased, the calculated difference between the two devices also increased, resulting in wide 95% LOA (−5.74 to 9.62 µm) spanning 15.36 µm, indicating poor agreement at an individual subject level (subgroup analyses presented in Table 2 and Figure 4).

**Table 2:** Retinal Nerve Fiber Layer Thicknesses and Total Macular Volume Among Cirrus HD-OCT vs. Spectralis Study Participants.

	Cirrus-HD OCT	Spectralis SD-OCT	r	*P value*	Mean Difference (95% CI)	Lower LOA (95% CI)	Upper LOA (95% CI)	Range
RNFL Thickness*								
All Participants (n = 95)	90.6±12.0	92.6±13.6	0.96	<0.001	1.94 (1.15 to 2.72)	−5.74 (−7.09 to −4.39)	9.62 (8.27 to 10.97)	15.36
RRMS (n = 61)	88.2±11.7	89.4±13.1	0.96	<0.001	1.13 (0.19 to 2.06)	−6.18 (−7.80 to −4.56)	8.44 (6.82 to 10.06)	14.62
Healthy Controls (n = 34)	94.9±11.6	98.3±12.8	0.96	<0.001	3.39 (2.06 to 4.71)	−4.19 (−6.48 to −1.90)	10.97 (8.68 to 13.26)	15.16
TMV**								
All Participants (n = 98)	7.92±0.47	8.31±0.49	0.93	<0.001	0.387 (0.349 to 0.424)	0.013 (−0.052 to 0.078)	0.761 (0.696 to 0.826)	0.748
RRMS (n = 63)	7.78±0.47	8.17±0.50	0.93	<0.001	0.391 (0.346 to 0.437)	0.030 (−0.049 to 0.109)	0.752 (0.673 to 0.831)	0.722
Healthy Controls (n = 35)	8.16±0.35	8.54±0.39	0.86	<0.001	0.379 (0.310 to 0.447)	−1.022 (−0.141 to 0.097)	0.780 (0.661 to 0.899)	0.802

Abbreviations: OCT: Optical coherence tomography; RNFL: Retinal nerve fiber layer; RRMS: Relapsing remitting multiple sclerosis; TMV: Total macular volume; CI: confidence interval; LOA: limit of agreement.

*All measurements are in µm.

**All measurements are in mm^3^.

Across the cohort (n = 98), there was a strong correlation between the two machines for measurements of TMV (r = 0.93; P<0.001) (Figure 3). Spectralis OCT generally measured a greater TMV than Cirrus HD-OCT (Figure 4), where the average mean difference for TMV between the two devices was 0.387 mm^3^, and the 95% LOA (0.013 mm^3^ to 0.761 mm^3^) spanned 0.748 mm^3^, indicating fair agreement on average for TMV across the cohort, as well as at an individual subject level (subgroup analyses presented in Table 2 and Figure 4).

**Figure 5:**
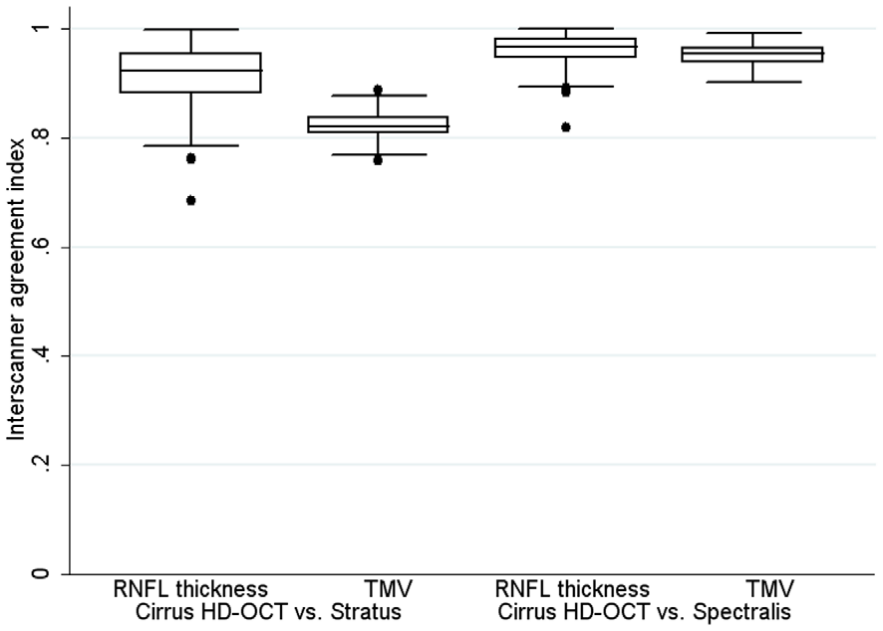
Boxplots of interscanner agreement for measures of RNFL thickness and TMV between Stratus OCT and Cirrus HD-OCT and Spectralis OCT and Cirrus HD-OCT. Boxplots illustrating the inter-quartile range for interscanner agreement indices show stronger agreement between Cirrus HD-OCT and Spectralis OCT measurements than between Cirrus HD-OCT and Stratus OCT. The lower and upper boundaries of each rectangle represent the 25^th^ and 75^th^ percentiles respectively, while the horizontal lines in between these represent the 50^th^ percentile.

The interscanner agreement between Cirrus HD-OCT and Stratus was 91.4±5.4% for RNFL thickness (n = 229) and 82.1±2.1% for TMV (n = 229) (Table 3). Meanwhile, the interscanner agreement between Spectralis and Cirrus HD-OCT was 96.1±3.0% for RNFL thickness (n = 95) and 95.1±2.0% for TMV (n = 98) (Table 3). Boxplots of the interscanner agreement indices for these comparisons are shown in Figure 5. Interscanner agreement remained virtually unchanged when the above analyses were repeated without inclusion of healthy controls.

**Table 3:** Interscanner Agreement Indices for Retinal Nerve Fiber Layer Thickness and Total Macular Volume measurements Among Cirrus HD-OCT vs. Stratus and Cirrus HD-OCT vs. Spectralis Study Participants.

Comparison	Median agreement (%)	Mean agreement (%)	SD of agreement (%)	Range of agreements (%)
Cirrus vs. Stratus RNFL thickness (n = 229)	92.3	91.4	5.4	68.5–99.9
Cirrus vs. Stratus TMV (n = 229)	82.1	82.1	2.1	75.8–88.6
Spectralis vs. Cirrus RNFL thickness (n = 95)	96.8	96.1	3.0	81.8–100
Spectralis vs. Cirrus TMV (n = 98)	95.4	95.1	2.0	90.2–99.1

Abbreviations: OCT: Optical coherence tomography; RNFL: Retinal nerve fiber layer; TMV: Total macular volume; SD: Standard deviation.

## Discussion

Results of this study have several important implications relevant to the utilization and interpretation of outcome measures from different OCT devices in clinical trials and at an individual patient level for clinical purposes. The Bland-Altman statistical method is useful for determining how much one method of measurement differs from an existing method of measurement [49], [50]. The mean difference between measurements indicates the level of agreement on average for the measurement of interest between the two devices across the cohort, while the limits of agreement (LOA) indicate the level of agreement for the measurement of interest between the devices at an individual patient or subject level. Clinical judgment must be applied to the interpretation and estimation of level of agreement of the mean differences or LOA generated by the Bland-Altman approach.

Compared to Stratus (TD-OCT), Cirrus HD-OCT (SD-OCT) averaged lower measures of RNFL thickness (mean difference: 7.4 µm) and higher measures of TMV (mean difference: −1.30 mm^3^), in keeping with previous studies of healthy controls, MS and glaucoma patients [45], [56], [57]. These results suggest poor agreement on average for these measures across the cohort. For this reason, we do not recommend using pooled results of RNFL thickness or TMV from these two devices as outcome measures in clinical trials. Although the LOA for TMV were narrow, the LOA for RNFL thickness were wide (23 µm) suggesting these two devices should also not be used inter-changeably to monitor RNFL thickness clinically. Further, given the correlation line for RNFL thickness between Cirrus and Stratus devices diverges from the line of equality, developing a formula allowing extrapolation of RNFL measures from one device to the other is likely to be difficult and potentially inaccurate.

Likewise, the correlation line for RNFL thickness between Cirrus HD-OCT and Spectralis (both SD-OCT devices) crosses the line of equality making the development of a formula allowing extrapolation of RNFL measures from one of these devices to the other difficult and also inaccurate. However, Spectralis averaged narrowly higher measures of RNFL thickness (mean difference: 1.94 µm) and TMV (mean difference: 0.387 mm^3^) than Cirrus HD-OCT, in keeping with prior studies of healthy controls, MS and glaucoma patients [58]–[60], suggesting excellent agreement on average for these measures across the cohort. Furthermore, the interscanner agreement indices between Spectralis and Cirrus HD-OCT were higher than those determined between Stratus and Cirrus HD-OCT, and had a median value of 96.8% for RNFL thicknesses and 95.4% for TMV (Table 3). For reference, a previous study found the overall median interscanner agreement was 91.1% for MRI measurements of T2-weighted lesion load, and 96.7% for measurements of T2-weighted lesion load on MRI scanners of the same field strength [51]. Thus, the agreement between SD-OCT machines examined in this study are comparable to the agreements noted between different MRI platforms that have been successfully co-utilized in previous multiple sclerosis trials [51]. It is unclear, however, if interscanner agreement for MRI measures representative of neurodegeneration including T1-weighted lesion load, T1-weighted lesion volume and cerebral atrophy are comparable to those for T2-weighted lesion load outlined above. Further studies assessing this are warranted. Nonetheless, the small mean difference and good interscanner agreement indices seen between the two SD-OCT devices included in this study imply pooled results of RNFL thickness and TMV from these devices could be used as outcome measures in clinical trials, provided patients were scanned on one device throughout the study.

In order to determine the acceptable mean difference between devices, two known factors were considered during the interpretation of the data. First, it is recognized from longitudinal analyses of OCT in MS, of predominantly the relapsing-remitting subtype, that on average each year of follow-up is associated with a 2 µm reduction in RNFL thickness, in the absence of clinical AON events [38]. Although the degree of RNFL change over time in active MS has not been specifically determined, we postulate it may be even greater in subsets of patients with inflammatory disease activity. This may be particularly relevant since MS patients with active MS tend to be targeted for recruitment to clinical trials and trials may be greater than one year in duration. Secondly, AON has been proposed as a disease model within which to study potential neuroprotective/neurorestorative agents, with OCT measures representing primary outcome measures. Following AON, 75% of patients will sustain a 10–40 µm reduction in RNFL thickness in the affected eye within 3–6 months (indicating significant and rapid axonal degeneration secondary to inflammation and demyelination) [27]. Given these two models for clinical trials, the expected difference between study groups is larger than the mean difference observed for the two spectral domain devices included in this study. As such, the mean difference in RNFL measurements between these spectral domain devices suggests excellent agreement on average across the cohort, and the two devices may be used together within a clinical trial. However, it is worth noting that based upon the two models for clinical trials outlined above, a larger sample size is likely to be required for longitudinal studies of MS patients in the absence of AON, given annualized reductions in RNFL thickness in these MS patients are less than those seen over short periods of time following AON. Based upon the wide LOA, however, if both Cirrus HD-OCT and Spectralis OCT were to be utilized to generate pooled measures of RNFL thickness or TMV in clinical trials, it would be imperative that a patient scanned on one device continue to be scanned on that same device for the duration of the study. Since the LOA for RNFL thickness were wide (15.41 µm) between the spectral domain devices analyzed, this indicates poor agreement at an individual subject level, suggesting these two devices should not be used inter-changeably to monitor RNFL thickness clinically. Clinicians should be aware of the poor agreement between Cirrus HD-OCT and Spectralis (as well as between Cirrus HD-OCT and Stratus) for RNFL thickness at an individual patient level and ideally perform scans on two machines for a period while switching from one OCT platform to another in order to allow equivalent comparison retrospectively and prospectively.

Differences in the segmentation algorithms between the OCT devices may explain some of the observed differences in measures of TMV between the devices [43], [44], [61]–[63]. For example, retinal thickness is measured from the inner limiting membrane (ILM) to the inner/outer photoreceptor junction with Stratus, from the ILM to the retinal pigment epithelium layer with Cirrus HD-OCT, and from the ILM to Bruch's membrane with Spectralis. These differences may explain why Stratus measures the lowest TMV measures and Spectralis the highest TMV measures.

Differences in resolution and image acquisition speeds between the investigated OCT devices may also explain some of the observed differences in retinal measures [39]–[42], [64]. Acquisition rates (axial scans per second) and resolution are 400 and 10 µm respectively with Stratus (TD-OCT), 27,000 and 5 µm respectively with Cirrus HD-OCT (SD-OCT), and 40,000 and 3.9 µm respectively with Spectralis (SD-OCT). The 70-fold increase in speed with SD-OCT technology allows greater sampling of retinal information and the generation of 3D images. Since less information is captured with TD-OCT, scan information must be averaged for retinal quantification, and only 2D images are obtained. There are additional differences in the processing techniques of these devices which may also contribute to observed discrepancies in retinal measurements. Cirrus HD-OCT software automatically centers on the optic nerve head and the fovea during RNFL and TMV measurement, while with Stratus OCT, the operator manually performs this task. There is also a time delay between data acquisition capture and the displayed fundus image with Stratus, while Cirrus-HD OCT and Spectralis capture data in real-time. Further, Spectralis utilizes a real-time eye tracking system that is able to adjust for eye movements during scanning. Despite these differences, our findings support suggestions that both TD-OCT and SD-OCT technologies capture RNFL thinning and TMV reduction in eyes of patients with MS [25], [45], [65]. However, it must be noted this was not the primary objective of this study, and as a result the healthy controls and MS participants in this study were not age-matched. It is important to emphasize that three commonly used OCT devices were assessed in terms of their agreement in this study; however, there are several other OCT devices which were not included in this study as they are not available at our institution. If other investigators are considering co-utilizing other OCT devices in clinical trials, it is imperative they first determine the agreement between these devices, rather than generalizing our findings as applicable to all OCT machines. Our finding that Cirrus HD-OCT and Spectralis (both SD-OCT) retinal measures could be potentially pooled as outcome measures may not necessarily imply acceptable agreement between other SD-OCT devices for this purpose. As data in this study were acquired from two separate cohorts (Stratus-Cirrus HD-OCT and Spectralis-Cirrus HD-OCT cohorts), it was not possible to assess agreement between Stratus and Spectralis by Bland-Altman analyses. However, prior studies assessing this demonstrate similarly poor agreement between these two devices, comparable to that observed in our Stratus-Cirrus HD-OCT cohort [66], [67]. Although not determined during this study, but relevant to the findings of this study, our group and others have previously demonstrated excellent intrascanner reproducibility for the OCT devices examined. Our group has found Stratus to have an interrater intraclass correlation (ICC) of 0.89, an intrarater ICC 0.98 and an intervisit ICC of 0.91 [36], while some other groups have reported even higher ICCs for Stratus [68]. Our group has also found Cirrus HD-OCT to have high ICCs, with an interater ICC of 0.97, an intrarater ICC of 0.99 and an intervisit ICC of 0.97 [37]. Similarly, Spectralis has also been shown to have a high intrarater ICC of 0.99 [69].

Trials of potentially remyelinating and neuroprotective drugs are forthcoming in MS. These studies are likely to be multi-center trials in order to enable the recruitment of appropriate numbers of patients to achieve adequate statistical power. The results of this study are relevant to the design and interpretation of studies utilizing OCT for outcome measures, particularly those in which there may be differences in the local availability of OCT technologies across centers in the study. We suggest Stratus TD-OCT should not be co-utilized with either Cirrus HD-OCT or Spectralis SD-OCT. Cirrus HD-OCT and Spectralis (both SD-OCT) may be co-utilized, provided patients scanned on one machine continue to be scanned on that same machine for the duration of the study, since this may improve data reliability consistent with the known excellent same-machine reliability of these devices. Thus for example, a center with only Cirrus HD-OCT and another center with only Spectralis may both participate in such a study. We suggest an equal number of centers with each device should be recruited for study enrolment and an equal number of participants should be recruited at each center, as is the manner in which commonly available MRI platforms have been successfully incorporated into prior MS clinical trials [53]–[55]. We hope our findings increase the ease of utilizing OCT for potential primary outcome measures in clinical trials as we embark upon the highly anticipated next chapter of MS research in remyelination and neuroprotection.
